# Editorial: Cellular and Molecular Mechanisms of *Mycobacterium tuberculosis* Virulence

**DOI:** 10.3389/fcimb.2019.00331

**Published:** 2019-10-09

**Authors:** Jianjun Sun, Patricia A. Champion, Fabiana Bigi

**Affiliations:** ^1^Department of Biological Sciences, Border Biomedical Research Center, University of Texas at El Paso, El Paso, TX, United States; ^2^Department of Biological Sciences, University of Notre Dame, Notre Dame, IN, United States; ^3^Institute of Biotechnology, National Institute of Agricultural Technology, Buenos Aires, Argentina

**Keywords:** *Mycobacterium tuberculosis*, drug resistance, virulence, pathogenesis, host-pathogen interaction

## Introduction

*Mycobacterium tuberculosis* (*Mtb*) is the bacterial pathogen that causes the majority of human tuberculosis (TB), the leading infectious disease in the world (Glaziou et al., 2018). *Mtb* invades the human host by aerosol and establishes infection in the lung by using virulence factors to combat host immunity. Over the past several decades, significant progress has been made in our understanding of *Mtb* pathogenesis. However, the mechanisms of *Mtb* virulence remain largely unknown. Moreover, the emergence of multidrug-resistant *Mtb* strains and co-infection of *Mtb* with HIV have posed new challenges in TB control. There is an urgent need to enhance our understanding of *Mtb* pathogenesis and to develop effective countermeasures against TB. This *Frontiers* Research Topic reports recent new findings that cover diverse aspects of cellular and molecular mechanisms of *Mtb* virulence.

### A New Role of the Well-Known Virulence Factor ESAT-6 in Regulating Macrophage Differentiation

ESAT-6 (6-kDa early secreted antigenic target), a well-documented *Mtb* virulence factor, is essential for *Mtb* pathogenesis, including phagosomal rupture, mycobacterial cytosolic translocation and cell-to-cell spreading (Hsu et al., 2003; Stanley et al., 2003; Abdallah et al., 2007; van der Wel et al., 2007; Houben et al., 2012; Manzanillo et al., 2012; Simeone et al., 2012, 2015; Zhang et al., 2016). ESAT-6 appears to function as an important modulator of host inflammatory responses by manipulating several intracellular signaling pathways in macrophages, T cells, and epithelial cells (Tsao et al., 1999; Giacomini et al., 2001; Junqueira-Kipnis et al., 2006; Pathak et al., 2007; Koo et al., 2008; Kurenuma et al., 2009; Mishra et al., 2010; Samten et al., 2011; Wong and Jacobs, 2011; Wu et al., 2019). Here, Refai et al. report a new role of ESAT-6 in macrophage differentiation and polarization. They found that during early infection, ESAT-6 induced differentiation of M0 and M2 macrophages toward the pro-inflammatory M1 phenotype to promote granuloma formation. Subsequently, ESAT-6 drove the phenotype switch from M1 to anti-inflammatory M2 macrophages to maintain the infection during the later persistent phases.

### New Mycobacterial Factors Important for Virulence

#### RD4

A number of regions of difference (RD) among mycobacterial species have been identified by comparative genomic studies (Mahairas et al., 1996; Behr et al., 1999; Gordon et al., 1999; Brodin et al., 2002; Lewis et al., 2003). RD1, which is present in the *Mtb* complex and in a related species *Mycobacterium marinum*, but absent from the *Mycobacterium bovis* Bacille Calmette–Guérin (BCG) genome, encodes an ESX-1 type VII secretion system that has been extensively investigated as a major virulence factor (Simeone et al., 2009; Tiwari et al., 2019). However, other regions of difference between mycobacterial pathogens and attenuated BCG strain have been characterized to a lesser extent. Ru et al. investigated the potential role of RD4 in virulence. RD4 is larger in *M. marinum* than in *Mtb*, but absent in *M. bovis*, including BCG, suggesting a gradual decay of RD4 in mycobacterial genomes in the order of *M. marinum, Mtb*, and *M. bovis*. The knock-in strains of BCG and *M. marinum* containing the entire or partial RD4 regions exhibited alterations of wild-type virulence in both mouse and zebrafish models of infection. Thus, RD4 appears to be a new locus contributing to the mycobacterial virulence.

#### CitE

Bacterial citrate lyase, which is important for both metabolism and virulence, is composed of three subunits, CitD (γ), CitF (α), and CitE (β) (Griffiths et al., 2012; Torres et al., 2012). The *Mtb* genome encodes 2 paralogous CitE subunits (CitE1 and CitE2), but their role in *Mtb* virulence has not been explored. Arora et al. biochemically and functionally characterized the CitE enzymatic subunits. The purified CitE1 and CitE2 proteins degraded acetyl-CoA and propionyl-CoA *in vitro* and the genes encoding both enzymes were up-regulated when *Mtb* was exposed to oxidative stress. Moreover, deletion of the *citE* genes from the *Mtb* genome reduced the resistance to oxidative stress, intracellular replication in macrophages, and growth in a guinea pig infection model. This study suggests that CitE may be a potential target for TB drug development.

#### A Novel Phylogenetic Clade Associated Hypervirulent Strain

Rajwani et al. analyzed the phylogenetic relatedness of a hypervirulent *Mtb* strain (H112) with a global collection of *Mtb* genomes and identified a novel phylogenetic clade that share single-nucleotide polymorphisms (SNPs) in key virulence-associated loci, including the *mce1* locus and the *phoP* gene. This clade includes four hypervirulent strains isolated from geographically diverse regions. The common SNPs and structural variations within the clade may be considered as potential genetic determinants of hypervirulence for future studies.

### The Host Factors Affected by *M. bovis* Infection

While *Mtb* is the most common cause of human TB, *M. bovis* can cause TB in both humans and cattle, making it a zoonotic threat to both food safety and public health (Cosivi et al., 1998; Renwick et al., 2007; Michel et al., 2010). Moreover, the knowledge obtained in the studies of *M. bovis* infection is valuable for understanding of *Mtb* infection due to their close relationship. In the comparative proteomic study done by Li et al., they identified proteins that were differentially regulated in human macrophages following infection with *M. bovis*, including proteins in several pathways that are similar to *Mtb* infections, such as the phagosome maturation pathway and the TNF signaling pathway. In addition, in a number of proteins and enzymes that are mainly involved in metabolic pathways, endocytosis and endosome trafficking events were found to be uniquely affected by *M. bovis* infection.

### New Insights Into the Drug-Resistant Mechanisms

Drug resistance is mainly caused by mutations in the *Mtb* genome, particularly by single-nucleotide polymorphisms in genes whose protein products are directly targeted by anti-TB drugs (Coculescu, 2009; Stucki and Gagneux, 2013). Hameed et al. provided a comprehensive review on the major molecular targets that are related to drug resistance mechanisms of *Mtb*.

The mutations in the *thyA* (encoding thymidylate synthase A) and *folC* (encoding FolC-dihydrofolate synthase) genes have been associated with resistance to para-aminosalicylic acid (PAS; Rengarajan et al., 2004; Zhao et al., 2014; Meumann et al., 2015), a second-line anti-TB drug. Methionine is structurally related to anti-folate drugs and is shown to antagonize PAS. However, the mechanism for methionine-based antagonism remains undefined. Using both targeted and untargeted approaches, Howe et al. found that MetM, a putative amino acid transporter, plays a crucial role in the synthesis of folate precursors, which antagonizes PAS activity.

Drug induced reversion of antibiotic resistance has drawn recent attention as a prospective approach to combat drug resistance (Baym et al., 2016). FS-1, a new anti-TB drug, induces antibiotic resistance reversion in *Mtb*. In the report done by Ilin et al., FS-1 was used in combination with standard anti-TB antibiotics on guinea pigs infected with an XDR-*Mtb* strain. The genetic changes in *Mtb* genomes following infection were analyzed and FS-1 was found to cause a counter-selection of drug-resistant variants that sped up the recovery of the infected animals from XDR-TB. While the drug resistance mutations remained intact in more sensitive isolates, reversion of drug resistance was associated with a general increase in genetic heterogeneity of the *Mtb* population.

## Conclusions

The articles in this Research Topic present new findings regarding the cellular and molecular mechanisms of *Mtb* virulence, including characterization of new roles for known virulence factors, identification of new virulence factors, and the elucidation of drug-resistance mechanisms and reversion. This Research Topic, together with many recent publications, enhances our understanding of the mechanism of *Mtb* virulence and pathogenesis.

## Author Contributions

JS, PC, and FB have made a substantial, direct and intellectual contribution to the work, and approved it for publication.

### Conflict of Interest

The authors declare that the research was conducted in the absence of any commercial or financial relationships that could be construed as a potential conflict of interest.
